# A New Method of Using Sensor Arrays for Gas Leakage Location Based on Correlation of the Time-Space Domain of Continuous Ultrasound

**DOI:** 10.3390/s150408266

**Published:** 2015-04-09

**Authors:** Xu Bian, Yu Zhang, Yibo Li, Xiaoyue Gong, Shijiu Jin

**Affiliations:** State Key Laboratory of Precision Measurement Technology and Instrument, Tianjin University, Tianjin 300072, China; E-Mails: bx332@tju.edu.cn (X.B.); slyb@tju.edu.cn (Y.L.); juliayue1208@gmail.com (X.G.); shjjin@tju.edu.cn (S.J.)

**Keywords:** continuous ultrasound, gas leakage, location, real time, dispersion, sensor array

## Abstract

This paper proposes a time-space domain correlation-based method for gas leakage detection and location. It acquires the propagated signal on the skin of the plate by using a piezoelectric acoustic emission (AE) sensor array. The signal generated from the gas leakage hole (which diameter is less than 2 mm) is time continuous. By collecting and analyzing signals from different sensors’ positions in the array, the correlation among those signals in the time-space domain can be achieved. Then, the directional relationship between the sensor array and the leakage source can be calculated. The method successfully solves the real-time orientation problem of continuous ultrasonic signals generated from leakage sources (the orientation time is about 15 s once), and acquires high accuracy location information of leakage sources by the combination of multiple sets of orientation results. According to the experimental results, the mean value of the location absolute error is 5.83 mm on a one square meter plate, and the maximum location error is generally within a ±10 mm interval. Meanwhile, the error variance is less than 20.17.

## 1. Introduction

Gas leakages in pressure systems are serious faults that can affect the tightness of vacuum structures, reduce the system operational safety coefficient, and can cause economic losses. Thus, a reliable real-time detection method to quickly identify the source of leakages is very necessary.

In recent years, the demand for real-time gas leakage location has increased yearly [1] and how to quickly and effectively locate leakage sources has become an urgent problem to be solved. According to different theories, current leakage detection technology mainly includes four methods: optical methods [2,3,4], the pressure change method [5], the resistance change method [6] and the acoustic emission (AE) method. The AE method detects the position of leakage holes by analysis of the leakage acoustic signal, which is collected by an AE sensor. Compared to the other methods, the AE method is easy to implement, the structure of the detected object does not need to be changed, it has fast location speed, and high immunity from interference. However, the ultrasonic leakage signal is a continuous signal without time domain features and the propagation characteristics are complicated [7,8,9,10,11], thus the position cannot be determined using the traditional Time Difference of Arrival (TDOA) technique [12]. Therefore, the traditional AE method presents some shortcomings in locating the continuous ultrasonic signal and it needs to be further explored. Mostafapour *et al.* [13] proposed a leak-locating algorithm in pressurized gas pipes based on a wavelet transform, filtering and cross correlation techniques; the error in leak location was less than 5%. Meng *et al.* [14] performed an acoustic experimental study on leak detection and localization for gas pipelines, conducted on a high-pressure and long-distance leak test loop. The researchers found that most acoustic leak signals were within the 0–100 Hz range, and they used different de-noising methods for different noise signals to improve the leakage location formula considering the pressure and temperature. Li *et al.* [15] used the cross-time–frequency spectra of leakage-induced acoustic vibrations to obtain the leak location in gas pipelines. Some ultrasonic leak detection equipment like UL101 [16,17] are used to locate leakages. The equipment locates the leakage source by collecting the ultrasonic leakage signal from the air surrounding the leakage holes. However, its detection range is small, and the equipment needs to be manually scanned in every suspicious area, so the method is time-consuming. Sedlak *et al.* [18] compared the first-arrival determination results for thin plates obtained from a two-step AIC picker with Kurz’s method, STA/LTA method, and standard threshold crossing technique. Kitajima *et al.* [19] determined the leakage source position by considering that the AE signal attenuates with the distance. However, this method is easily affected by the structure of the detected object and background noise, so it has a large location error under normal circumstances. Holland *et al.* proposed an 8 × 8 sensor array to collect ultrasonic signals of orbiting spacecraft leaks [20,21,22,23], and calculated the intensity distribution of the wave number diagram (k-domain) of the collected signal, to estimate the direction of the sound source. Meanwhile, two sets of array orientation results are used to locate the leakage. However, this method requires a large number of sensors in the array (at least 64), and the location accuracy is poor (the biggest location error is 20 mm in a one square meter plate), therefore its applications are limited.

This paper proposes a new method for the location of continuous leakage sound sources. This method analyzes the continuous gas-leakage generated ultrasonic signal, and creates a mathematical model of the continuous ultrasound signal that propagates in the plate. Meanwhile, when the continuous broadband ultrasound propagates in the plate, it also solves the problem of location results affected by the continuously changing sound velocity. Besides, through the time-space relativity of ultrasonic signals generated by the same leakage source, the method achieves the detection and location of the gas leakage source. A large amount of experimental results indicate that this algorithm can stably locate leakage sources in real-time. Compared with the previous location method [20,21,22], it can achieve higher location accuracy with a smaller number of sensors in one array, and has better real-time detection properties.

## 2. Location Method

**Figure 1:**
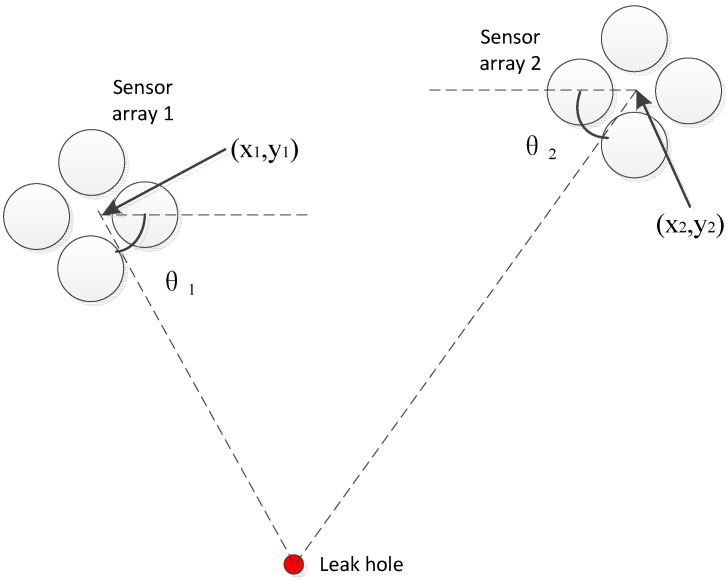
Locating principle diagram.

In practice, the ultrasonic signal generated by the same leakage source is continuous and stable, thus a sensor array can be used for the signal direction. Moreover, the leakage location can be obtained by combining multiple sets of results from sensor arrays oriented in different positions. Figure 1 shows the location principle diagram.

In Equation (1), the parameters (x_1_,y_1_), (x_2_,y_2_) are known, thus to obtain the source position (x,y), θ_1_ and θ_2_ are necessary:

(1) $$\begin{array}{l} \;y-y_{1}=\tan(2\pi -\theta_{1})(x-x_{1})\\ y-y_{2}=\tan(\pi +\theta_{2})(x-x_{2}) \end{array}$$

However, it is difficult to obtain θ_1_ and θ_2_ using a continuous signal by the traditional method, so this needs to be further explored. According to the previous location method [20], the k-domain distribution of a sensor array can be acquired by using high precision space sampling, and then the directional information of the leakage source can be obtained. However, this method is limited by the spatial resolution requirements, thus the array requires a high number of sensors to achieve a higher orientation accuracy. 

The proposed method refers to the beamforming algorithm [24], to choose a sensor array with multiple sensors, and then collects leakage source ultrasonic signals synchronously to acquire a set of spatially coherent signals. This method can not only ensure that the multipath signals which are collected by the sensors of an array have a very similar degree of attenuation and distortion, but also can avoid the effect of leakage signal randomness. Moreover, from the dispersion equation, the relationship between signal frequency and sound velocity has been considered in the algorithm which can solve the influence of location results affected by the continuously changing sound velocity. Finally, by integrating the specific frequency band, the method seeks the angle that makes the multipath signals have the strongest correlation in the time-space domain, so as to estimate the direction of the leakage source and solve the location problem.

## 3. Algorithm Theory

This paper uses an L-type array to achieve a high of orientation accuracy in the 90° range, and achieves the fast location of the leakage source in any position by integrating multiple sets of L-type sensor array orientation results. The process of analysis and testing the variable array directional characteristics is widely discussed in [25,26,27].

**Figure 2:**
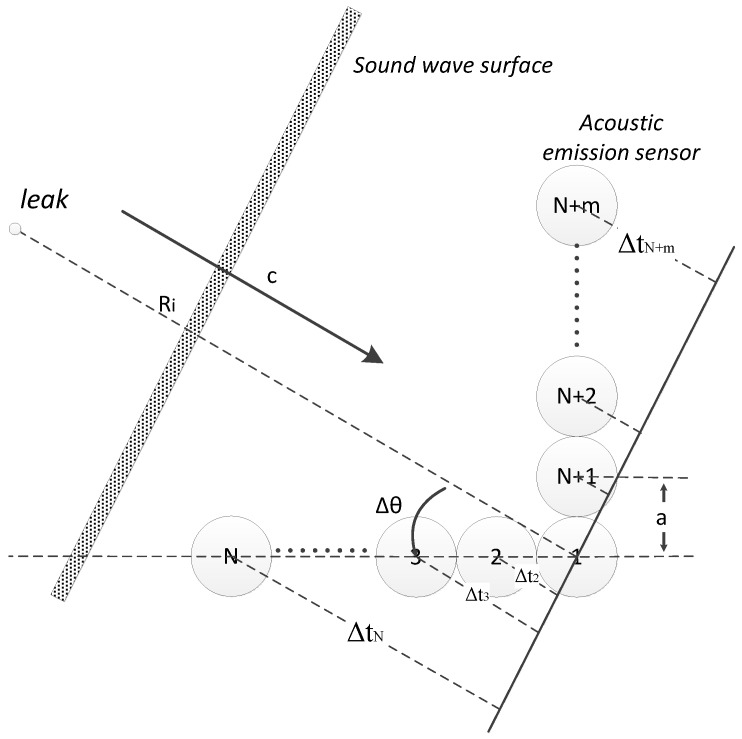
L-type sensor array.

Figure 2 shows the structure of the array. N + m represents the total amount of sensors in the array, where *N* and *m* are the number of sensors in the horizontal and vertical directions respectively; *a* represents the center-to-center distance between two equally-spaced sensors; *c* is the sound velocity; represents the angle between the sound source direction and the reference direction; *R_i_* is the distance between leakage hole and the *i*-th sensor.

According to experimental tests, the leakage ultrasound power is mainly concentrated within the 0–500 kHz frequency range, and the signal wavelength (λ) is approximately 1 cm. *R_i_* is usually more than 50 times longer than λ and the sound waveform can be considered as plane wave [28].

Due to the fact the leakage signal is broadband, the frequency dispersion phenomenon exists when that signal propagates in the thin plate [29]. Thus, the sound velocity *c* depends on the frequency *f* that continuously changes. According to the wave equation [29,30], the term *P_r,k_*(*t*) which represents the noise at the location of the reference sensor can be expressed as a function of the frequency in the k mode (Equation (2)). In this research, the first sensor is defined as the reference sensor. $A_{k}(f)$ is a random amplitude and frequency’s spectrum of the leak noise at the location of the reference sensor in the k mode. *f* represents the single frequency that varies within the range *f*_0_–*f*_n_:

(2) $$P_{r,k}(t)=\int_{f_{0}}^{f_{n}}A_{k}(f)\cdot \exp(2j\pi f\cdot t)df$$

According to the geometrical relationship, at a specific angle Δ*θ* and *c*, the signal from the *i*-th sensor has a certain arrival time difference $△t_{i}$ compared to the reference sensor. Because of the frequency dispersion，the sound velocity *c* is not unique and continuously changes according to frequency (*f*) changes in the k mode. Thus, the relationship $c_{k}(f)$ can be obtained by solving the dispersion equation. The V*t_i_* can be rewritten as:

(3) $$\begin{array}{l} △t_{i}(\Delta \theta ,f)=\frac{a}{c_{k}(f)}\Psi_{i}(\Delta \theta )\\ \Psi_{i}(\Delta \theta )=\{\begin{array}{l} \begin{array}{cc} i\cdot \cos\Delta \theta & i=1,2,\cdots ,N \end{array}\\ \begin{array}{cc} (i-N)\cdot \sin△\theta & i=N+1,N+2,\cdots N+m \end{array} \end{array} \end{array}$$

According to Equations (2) and (3), assuming the actual leakage direction is *θ*, the theoretical noise at the location of the *i*-th sensor is:

(4) $$P_{i,k}(t)=\alpha_{i}\cdot \int_{f_{0}}^{f_{n}}A_{k}(f)\cdot \exp[2j\pi f\cdot t+2j\pi f\cdot \frac{a}{c_{k}(f)}\Psi_{i}(\theta )]df$$

*α_i_* combines the distance- and frequency-dependent attenuation effect of geometric diffraction, material absorption, and radiation loss (into the air): 

Let:

(5) $$P_{r,f,k}(t)=A_{k}(f)\cdot \exp(2j\pi f\cdot t)$$

Thus, Equation (4) becomes:

(6) $$P_{i,k}(t)=\alpha_{i}\cdot \int_{f_{0}}^{f_{n}}P_{r,f,k}(t)\cdot \exp[2j\pi f\cdot \frac{a}{c_{k}(f)}\Psi_{i}(\theta )]df$$

In practice, the actual leakage direction *θ* is the information we want to obtain, and is unknown in advance, thus the variable Δ*θ* is introduced to obtain the *θ*. It means assuming the leakage direction is Δ*θ*, and then the noise *P_i,k_*(*t*) can be delayed in the opposite direction of the acoustic wave propagation.

(7) $$P_{i,k}（t,\Delta \theta ）=\alpha_{i}\cdot \int_{f_{0}}^{f_{n}}P_{r,f,k}(t)\cdot \exp\left\{2j\pi f\cdot \frac{a}{c_{k}(f)}\left[\Psi_{i}(\theta )-\Psi_{i}(\Delta \theta )\right]\right\}df$$

By superimposing every *P_i,k_*(*t*, Δ*θ*) to get the output of L-type array under the specific angle Δ*θ*, thus, *P_Σ,k_*(*t*, Δ*θ*) can be rewritten as:

(8) $$P_{\sum ,k}(t,\Delta \theta )=\sum_{i=1}^{M+m}\alpha_{i}\cdot \int_{f_{0}}^{f_{n}}P_{r,f,k}(t)\cdot \exp\left\{2j\pi f\cdot \frac{a}{c_{k}(f)}\left[\Psi_{i}(\theta )-\Psi_{i}(\Delta \theta )\right]\right\}df$$

The distance among each sensor of the array is very small, thus neglecting the differences of *α_i_*, assuming *α* = *α_i_*, and Equation (8) can be simplified as follows:

(9) $$P_{\sum ,k}(t,\Delta \theta )=\alpha \cdot \sum_{i=1}^{M+m}\int_{f_{0}}^{f_{n}}P_{r,f,k}(t)\cdot \exp\left\{2j\pi f\cdot \frac{a}{c_{k}(f)}\left[\Psi_{i}(\theta )-\Psi_{i}(\Delta \theta )\right]\right\}df$$

According to the Equation (3), Equation (9) can be expanded as:

(10) $$P_{\sum ,k}(t,\Delta \theta )=\alpha \cdot \int_{f_{0}}^{f_{n}}P_{r,f,k}(t)\cdot \left\{\sum_{i=1}^{N}e^{2j\pi f\cdot \frac{i\cdot a}{c_{k}(f)}\left[\cos(\theta )-\cos(\Delta \theta )\right]}+\sum_{i=N+1}^{N+m}e^{2j\pi f\cdot \frac{(i-N)\cdot a}{c_{k}(f)}\left[\sin(\theta )-\sin(\Delta \theta )\right]}\right\}df$$

Let:

(11) $$\{\begin{array}{c} q_{h}=\frac{2j\pi fa}{c_{k}(f)}[\cos(\theta )-\cos(\Delta \theta )]\\ q_{v}=\frac{2j\pi fa}{c_{k}(f)}[\sin(\theta )-\sin(\Delta \theta )] \end{array}$$

According to the properties of geometric progression and Euler’s formula, Equation (10) can be written as:

(12) $$P_{\sum ,k}(t,\Delta \theta )=\alpha \cdot \int_{f_{0}}^{f_{n}}P_{r,f,k}(t)\cdot \left\{e^{\left(-N-\frac{1}{2}\right)q_{h}}\cdot \frac{\sin(N\cdot q_{h})}{\sin(q_{h})}+e^{\left(-m-\frac{1}{2}\right)q_{v}}\cdot \frac{\sin(N\cdot q_{v})}{\sin(q_{v})}\right\}df$$

According property of function $\frac{\sin(n\cdot x)}{\sin(x)}$ and $e^{x}$, $P_{\sum ,k}(t,\Delta \theta )$ reaches the maximum when both qh and qv are zero, meanwhile, based on Equation (11), one obtain Δθ=θ,Δθ∈(0,90). In other words, the output of the array *P_Σ,k_*(*t*, Δ*θ*) is the maximum in the k mode assuming that the estimated direction is the same as the actual leakage source. 

In practice θ is unknown, but the signal at the location of the *i*-th sensor $P_{i}(t)$ can be acquired by an AE sensor. According to Equation (6):

(13) $$P_{i,f,k}(t)=P_{r,f,k}(t)\cdot \exp\left\{2j\pi f\cdot \frac{a}{c_{k}(f)}\Psi_{i}(\theta )\right\}$$

$P_{i,f,k}(t)$ represents $P_{i}(t)$ which the frequency is *f* in the k-th mode. $P_{i,f,k}(t)$ is made explicit in order to reduce the influence of frequency dispersion phenomenon to the computing results. For example, under the A0 vibrating mode, the speed variation of a 200 kHz broadband signal can reach 600 m/s. Thus, $P_{i}(t)$ can be represented by $P_{i,f,k}(t)$ as:

(14) $$P_{i}(t)=\sum_{k}\int_{f_{0}}^{f_{n}}P_{i,f,k}(t)df$$

By substituting Equation (13) into Equation (9), the output of array in the k mode is:

(15) $$P_{\sum ,k}(t,\Delta \theta )=\alpha \cdot \sum_{i=1}^{M+m}\int_{f_{0}}^{f_{n}}P_{i,f,k}(t)\cdot \exp[2j\pi f\cdot \frac{a}{c_{k}(f)}\Psi_{i}(\Delta \theta )]df$$

According to the Lamb theory [29], under the condition we considered (plate is less than 6 mm thick, and the signal frequency within the range 100–300 kHz), only two vibrating modes exist, the extensional mode A0 and the flexural mode S0. According to [31] the A0 mode has a greater contribution to the locating result than the S0 mode. The experimental tests conducted showed that the proposed method can achieve high accuracy location by only considering the single A0 mode, thus the contribution of the S0 mode can be neglected. Equation (15) can be written as:

(16) $$P_{\sum ,A_{0}}(t,\Delta \theta )=\alpha \cdot \sum_{i=1}^{M+m}\int_{f_{0}}^{f_{n}}P_{i,f}(t)\cdot \exp[2j\pi f\cdot \frac{a_{i}}{c_{A_{0}}(f)}\Psi_{i}(\Delta \theta )]df$$

In the Equation (16), *P_Σ,A_0__*(*t*, Δ*θ*) is a function of Δθ and *t*. Selecting the time window as (t_a_,t_b_) and integrating the Equation (16), the energy output of sensor array (E) can be obtained under the specific angle Δθ:

(17) $$E_{A_{0}}(\Delta \theta )=\alpha \cdot {\int_{t_{a}}^{t_{b}}\left\{\sum_{i=1}^{M+m}\int_{f_{0}}^{f_{n}}P_{i,f}(t)\cdot \exp[2j\pi f\cdot \frac{a_{i}}{c_{A_{0}}(f)}\Psi_{i}(\Delta \theta )]df\right\}}^{2}dt$$

**Figure 3:**
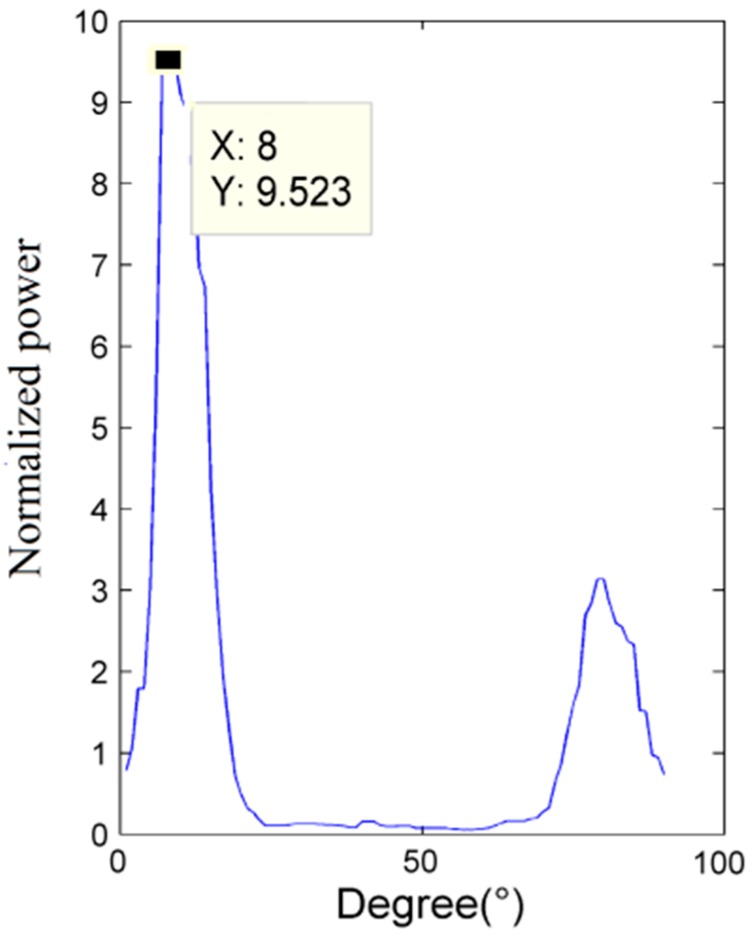
Angle-power relation.

According to Equation (12), when Δ*θ* = θ the estimated leakage direction and the actual one are the same and E reaches its maximum value. Figure 3 plots the relationship between the angle Δ*θ* and the normalized power E, calculated with numerical simulation using MATLAB^®^: the angle corresponding to the maximum power peak gives the estimated position of the acoustic source. 

**Figure 4:**
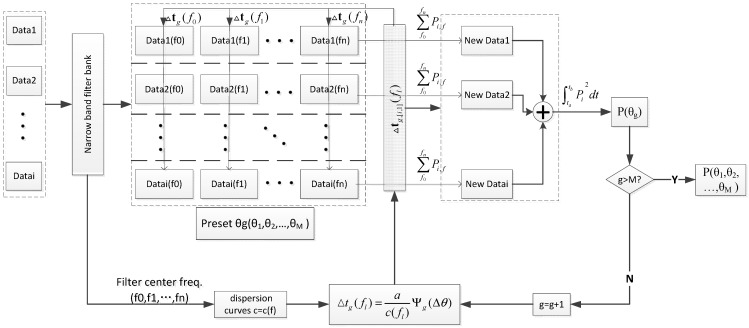
Up-to-date algorithm flow chart.

To take into account the conditions of the experimental test, a narrow band filter has been used to obtain the required band signal with a frequency band that is so narrow that the velocity of sound can be regarded as a unique value. Figure 4 represents the up-to-date flow chart.

## 4. Experimental Setup

### 4.1. Assembly of the Apparatus

**Figure 5:**
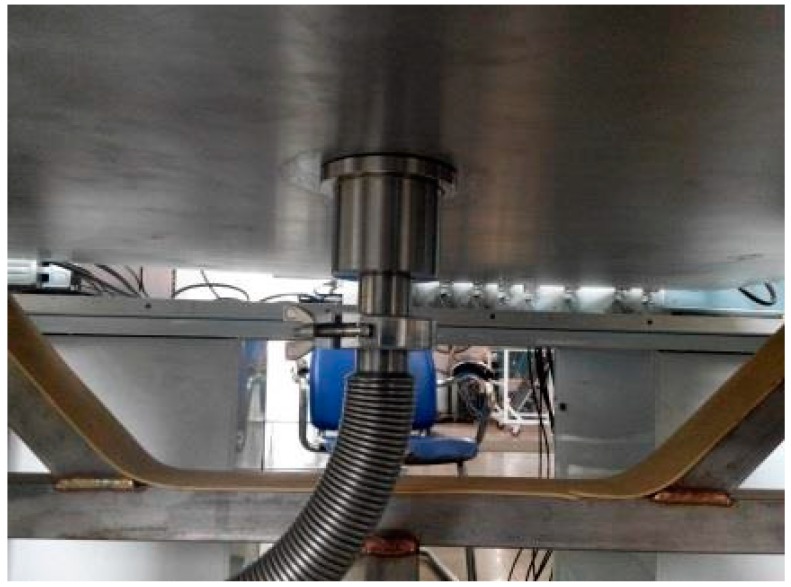
Gas leakage simulation.

**Figure 6:**
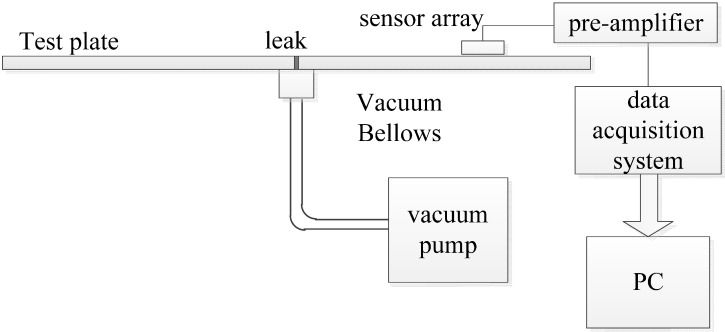
Experimental apparatus (schematic diagram).

Experimental tests have been carried out using a 302 stainless steel plate and a magnesium aluminum alloy plate. Both plates are square (1000 × 1000 mm) and 2.5 mm thick. A series of circular holes have been drilled randomly on the surfaces of the two plates in order to simulate leakage holes; the size of the holes varied within the range 0.8–2 mm (in diameter). Acoustic data from the sensor array has been acquired using a fully digital 16-channel recorder (DS-16A), at a sampling rate of 3 MHz, and sent to a PC. Saved experimental data have been processed with the MATLAB^®^ software. Vacuum grease ensures coupling between the sensors array and the plate. A pre-amplifier (gain set to 40 dB) is installed between the sensor array and the data acquisition system, to boost the signal and reduce the effects of noise and interference. A vacuum pump with a vacuum nozzle provided the loading, and thus the leakage pressure. The leakage hole is connected with the vacuum pump through the vacuum suction nozzle as shown in Figure 5. By starting the vacuum pump air is drawn off from the vacuum nozzle and a leakage source is simulated. The ultrasonic signal generated by the leakage can be detected and acquired. The ultrasonic signal attenuation is very low when it propagates in metal media, thus both resonance and scattering can occur [20]. Moreover, the environmental noise affects the low frequency signal. Thus, in order to avoid these interferences the 100–300 kHz frequency band is selected. Figure 6 shows the experimental apparatus.

Table 1 summarizes the main characteristics of the two plates.

**Table 1:** Plates’ main characteristics.

Material	Modulus of Elasticity E (KN/mm^2^)	Poisson’s Ratio σ	Density ρ (g·cm^−3^)
302 Stainless steel	210	0.305	7.93
Magnesium aluminum alloy	40	0.275	<1.8

**Figure 7:**
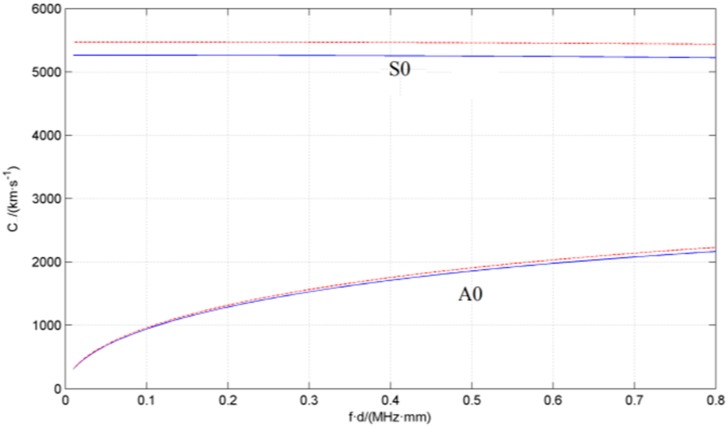
Dispersion curve of the experimental plates (A0: flexural mode, S0: extensional mode; Solid line: 302 steel, Dotted line: magnesium aluminum alloy).

The calculated values for the 302 stainless steel plate are 5826 m/s (c_1_) and 3115 m/s (c_s_) respectively. For the magnesium aluminum alloy plate, c_l_ is 5991 m/s, and c_s_ is 3266 m/s. By substituting these values into the dispersion equation [29], using the MATLAB^®^ software, the c(*f*) of the two materials can be calculated. The c(*f*) curves are shown in Figure 7.

Numerous experiments have been conducted on the two kinds of plate, and the orientation results are analyzed using A0 mode, S0 mode, and A0&S0 mode, respectively; the results are shown in Table 2. They indicate that the A0 mode has a greater contribution to the orientation result than the S0 mode, and a highly accurate result can be obtained by only considering the A0 mode alone, thus the S0 mode is neglected in the experiments.

**Table 2:** The orientation results in different modes.

Mode Used	302 Steel		Magnesium Aluminum Alloy	
	Mean Error (°)	Variance	Mean Error (°)	Variance
A0 mode	0.29	1.9039	0.1925	1.252178
S0 mode	6.04	388.8854	11.7425	700.4788
A0&S0 mode	0.28	3.1546	0.54	3.206737

### 4.2. Sizing of the Array

**Figure 8:**
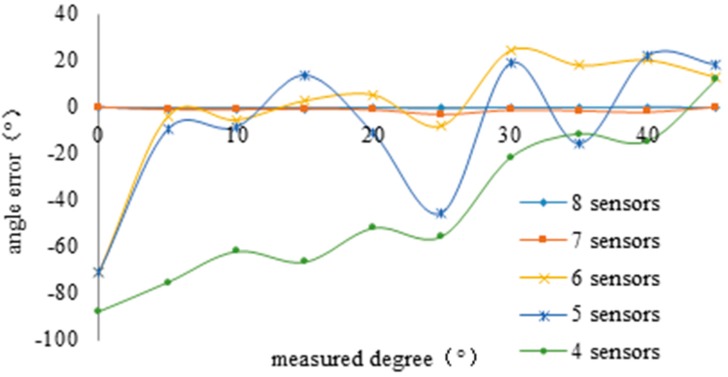
Relationship between number of sensors and accuracy.

According to Equation (11), the greater the number of sensors in an array, the higher the orientation accuracy that can be achieved. In order to find the relationship between the number of sensors and orientation accuracy, the array has been rotated one degree at a time to apply small changes to the relative angle between the leakage source and the array. For each rotation the data have been acquired. Figure 8 plots the curve of orientation errors depending on the position of the array with respect to the leakage source, for different numbers of sensors.

The tests show that the angle error increases sharply when the number of sensors in an array is less than seven. To take into account the demands of directional accuracy and robustness of the system, the L-type sensor array composed of eight AE sensors has been selected. Nano-30 (Physical Acoustics Co, Princeton, NJ, USA) sensors have been selected to ensure the best spatial response. Table 3 summarizes the main technical features of the sensors. 

**Table 3:** Sensor main technical features.

Item	Value
Peak Sensitivity, Ref V/(m/s)	62 dB
Peak Sensitivity, Ref V/μbar	−72 dB
Operating Frequency Range	125–750 kHz
Resonant Frequency, Ref V/(m/s)	140 kHz
Resonant Frequency, Ref V/μbar	300 kHz
Directionality	± 1.5 dB
Diameter	8 mm

**Figure 9:**
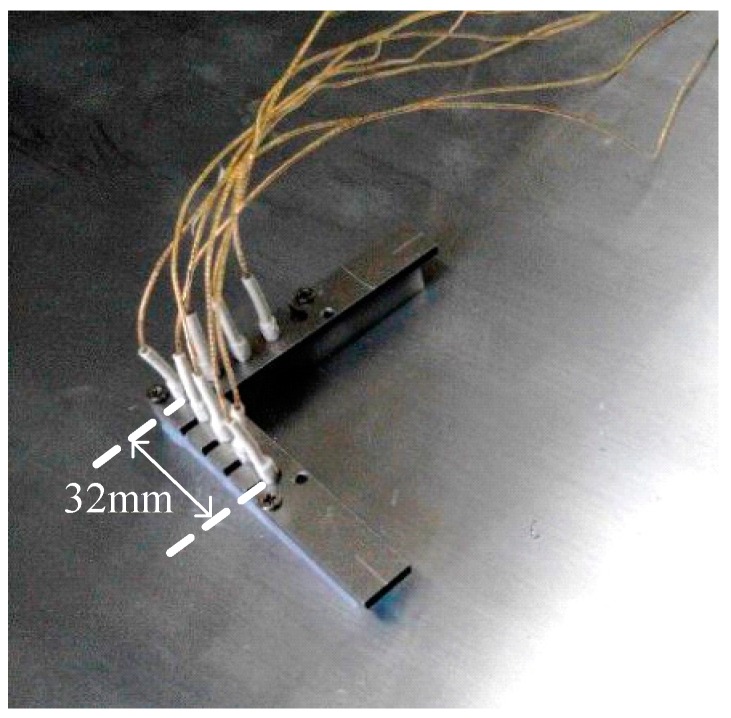
The 8-sensor array.

The sensor array is shown in Figure 9; the center-to-center distance between two adjacent sensors is 8 mm.

## 5. Results and Discussion

**Figure 10:**
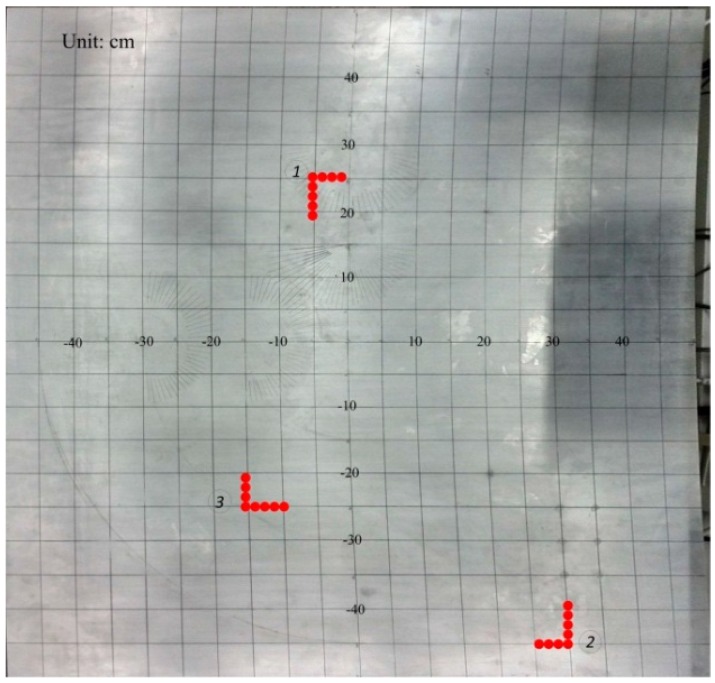
Sensor array positioning.

The accuracy of this method has been validated through experimental tests described in this section. A series of experiments have been performed with the sensor array at a variety of locations on the test plate to verify the accuracy of the proposed method. Take one condition as example, the leakage hole lies in the middle of the plate and its diameter is 1 mm; the sensor arrays have been placed onboard at three different positions, as shown in Figure 10.

**Figure 11:**
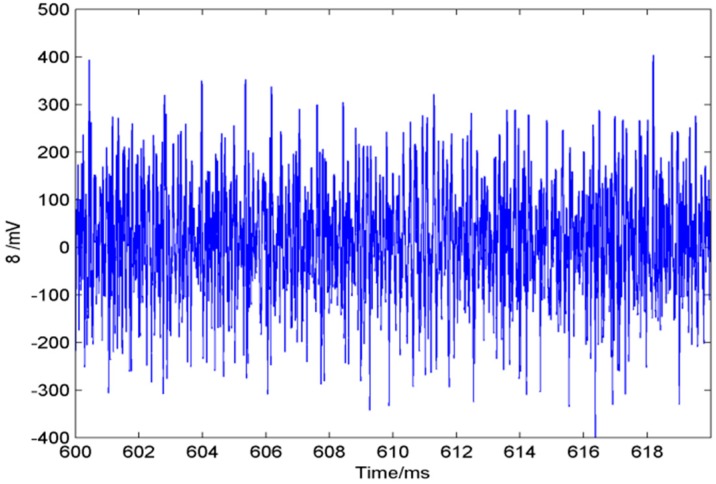
Acquired single-sensor signal (the time length is 0.01 s).

**Figure 12:**
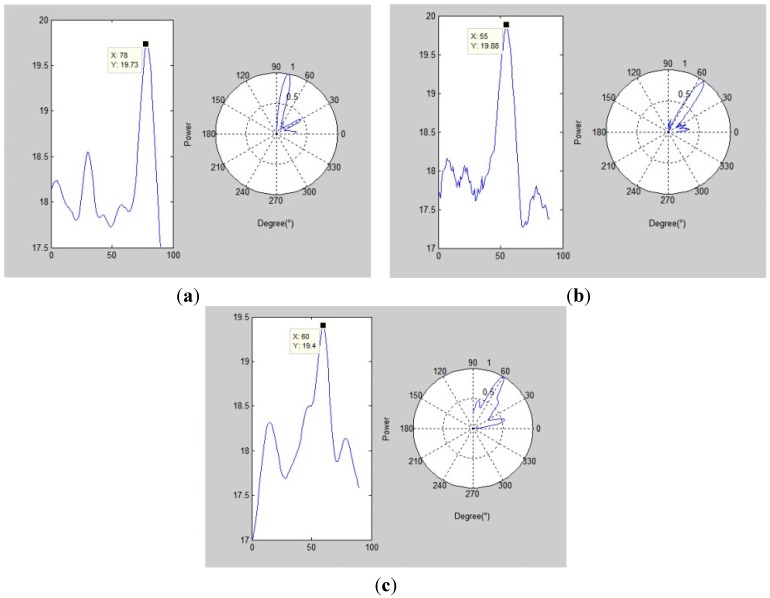
(**a**) Array position No.1; (**b**) Array position No.2; (**c**) Array position No.3.

The sensor array acquires the ultrasonic leakage signal using a 3 MHz sampling rate. Take one acquired single sensor signal in the array as the example (following Figure 11), while Figure 12 shows three calculated angle-power curves of the whole array’s signals in three different positions of the array on the plate, and the calculation method is mentioned in Section 3 (left: cartesian coordinate system; right: polar coordinate system).

The diagrams highlight that the angle-power curve shows a maximum peak for each position of the array, which corresponds to the leakage source direction. In order to avoid location errors, all the actual leakage angles have been measured using a high precision digital display protractor. For indicating the orientation accuracy of this method, several experimental data from different measurements were collected and analyzed using two different methods, *i.e.* the proposed method and the traditional method, which does not consider changes in sound velocity. Table 4 compares the orientation results obtained with the two methods.

**Table 4:** The orientation results comparison.

	Actual Leakage Angle (°)	Proposed Method without the Sound Velocity’s Changing (*c* = 2000 m/s)		Proposed Method	
		Mean Error (°)	Variance	Mean Error (°)	Variance
No.1 array	78.7	5.7	208.01	0	3.38
No.2 array	56.3	19.3	179.25	0.4	2.96
No.3 array	60	−13.1	152.77	−0.1	3.19

According to Table 4, the proposed method has a higher orientation accuracy, and the mean location error is within ±0.5°, while the single location error lies within ±2°. The comparison with the traditional method highlights that the proposed method gives a stable and high accuracy detection of the direction. The acoustic velocity error has a big influence on the results. Table 5 gives the location results from the orientation results (as shown in Table 4) of the three different position arrays. Error d represents the distance between the estimated leakage position and the actual leakage hole.

**Table 5:** Leakage location results.

	The Coordinate of Leakage Point(mm)	The Coordinate of Estimate Leakage Point (mm)	Error d (mm)
No.1 and No.2 array	(0,0)	(1.89,−9.67)	9.85
No.1 and No.3 array	(0,0)	(−1.64,8.00)	8.17
No.2 and No.3 array	(0,0)	(−5.51,1.22)	5.64
Comprehensive result	(0,0)	(−5.26,−0.45)	5.28

**Figure 13:**
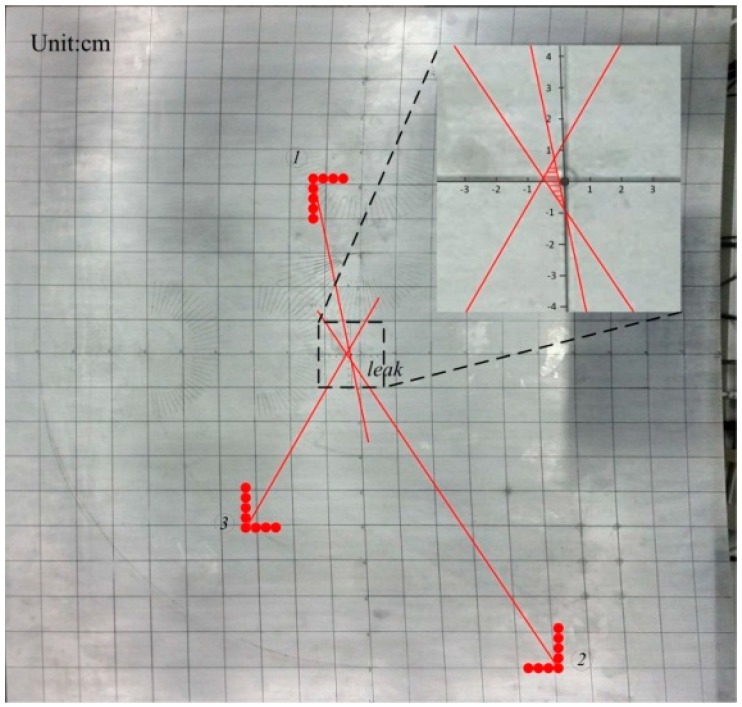
The estimated location area.

As mentioned in [20], based on three array orientation results, the leakage was located with an error of 10.6 mm on a 1 m^2^ plate. Under these conditions, the proposed algorithm gives an error of 5.28 mm. Meanwhile, the location error is 9.85 mm when the worse-case from a geometric perspective (the distance between the two sensor arrays and the leak hole are both longer than in [20] as mentioned) is considered. According to the results, the leakage location accuracy can be further improved by integrating multiple acquisitions of different arrays positions to estimate the location area (the shadowed area of Figure 13).

**Figure 14:**
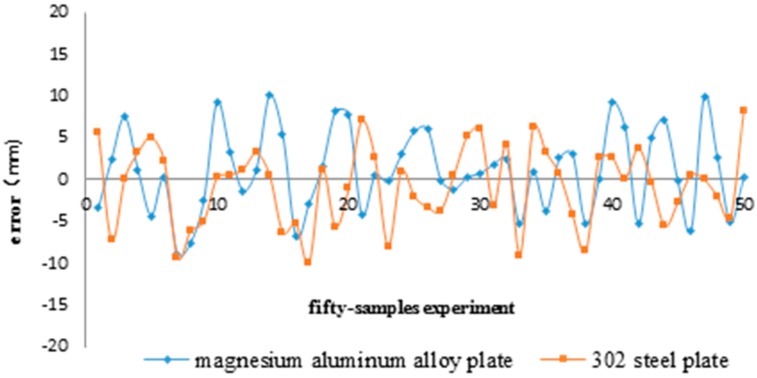
Position error.

Experimental tests confirmed that the results of this method are not affected by the array positions or the plate’s material. Two sensor arrays are placed at different positions randomly to detect the signal from any leakage hole on the same plate, and the plates made of two different materials have been tested. The experimental data are shown in Figure 14.

The location error is defined as the distance between the estimated leakage position and the actual leakage hole. According to Figure 14, the probability of the location error being less than 10 mm is 98%. The mean absolute value of error concerning the magnesium alloy plate is 3.42 mm, and the one for the steel plate is 5.66 mm; the variances are 24.4 and 22.2, respectively. These results are typical of many others we have obtained.

## 6. Conclusions

This research studied and analyzed the characteristics of continuous ultrasonic gas leakage signals. By solving the orientation problem, high accuracy localization of leakage holes is successfully achieved. Theoretical analysis and experimental verification of the orientation problem lead to the following conclusions:

(1) Experimental tests show that the leakage-generated acoustic emission signal is influenced by some factors such as media characteristics, leakage hole size, and sensor response. Moreover, the distortion which is introduced by the sensors cannot be neglected in order to achieve higher location accuracy.
(2) The leakage ultrasonic signal is a noise-like continuous broadband signal. According to the experimental results, the signal collected by an AE sensor is mainly in A0 mode (plate is less than 6 mm thick, and signal frequency within the range 100–300 kHz). Meanwhile, the S0 mode has an extremely small influence on the locating result, thus S0 can be neglected.
(3) This research presents a high-accuracy leakage source location method using fewer sensors to compose the sensor array. Moreover, the study solves the gas continuous leakage real-time localization problem based on the correlation of the signal in the time-space domain, which is generated from the leakage hole. Experimental results show that when the size of plate is 1000 × 1000 × 2.5 mm and the diameter of the leakage hole is larger than 0.8 mm, the mean location error is 5.83 mm, and the maximum location error is generally less than 10 mm. These results are typical of many others we have obtained. Therefore, this method provides a new approach to successfully solve the problem of real-time detection of gas leakages and location in large pressure vessels.

## References

[1] Murvay P.S., Silea I. (2012). A Survey on Gas Leak Detection and Localization Techniques. J. Loss Prevent. Process Ind..

[2] Gaunce M.T., Thompson D.R. (1997). Mir Photo/TV Survey (DTO-1118): STS-86 Mission Report.

[3] Graf J.C., Kittrell C., Arepalli S. (1999). Mir Leak Detection Using Fluorescent Tracer Gases. SAE Tech. Pap..

[4] Kroll A., Baetz W., Peretzki D. On Autonomous Detection of Pressured Air and Gas Leaks Using Passive IR-Thermography for Mobile Robot Application. Proceedings of the IEEE International Conference on Robotics and Automation.

[5] Lemon D.K., Friesel M.A., Griffin J.W., Skorpik J.R., Shepard C.L., Antoniak Z.I., Kurtz R.J. (1990). Technology Evaluation for Space Station Atmospheric Leakage.

[6] Fukushige S., Akahoshi Y., Koura T., Harada S. (2006). Development of perforation hole detection system for space debris impact. Int. J. Impact Eng..

[7] Al-Nasser Y.N., Datta S.K., Shah A.H. (1991). Scattering of Lamb waves by a normal rectangular strip weldment. Ultrasonics.

[8] Greve D.W., Tyson N, Oppenheim I.J. Interaction of defects with Lamb waves in complex geometries. Proceedings of the 2005 IEEE Ultrasonics Symposium.

[9] Ziola S.M., Gorman M.R. (1991). Source location in thin plates using cross-correlation. J. Acoust. Soc. Am..

[10] Beigelbeck R., Antlinger H., Cerimovic S., Clara S., Keplinger F., Jakoby B. (2013). Resonant pressure wave setup for simultaneous sensing of longitudinal viscosity and sound velocity of liquids. Meas. Sci. Technol..

[11] Kundu T., Nakatani H., Takeda N. (2012). Acoustic Source Localization in Anisotropic Plates. Ultrasonics.

[12] Lombard A., Zheng Y., Buchner H., Kellermann W. (2011). TDOA estimation for multiple sound sources in noisy and reverberant environments using broadband independent component analysis. IEEE Trans. Audio Speech Lang. Process..

[13] Davoodi S., Mostafapour A. (2014). Gas leak locating in steel pipe using wavelet transform and cross-correlation method. Int. J. Adv. Manuf. Technol..

[14] Meng L.Y., Wang W.C., Fu J.T. (2012). Experimental study on leak detection and location for gas pipeline based on acoustic method. J. Loss Prevent. Process Ind..

[15] Li S.Y., Wen Y.M., Li P., Yang J., Dong X.X., Mu Y.H. (2014). Leak location in gas pipelines using cross-time–frequency spectrum of leakage-induced acoustic vibrations. J. Sound Vib..

[16] Studor G. (2002). Ultrasonic Detectors in Space.

[17] Hoover A. (2002). Maryland Company Expanding Technology in Space-NASA Won’t Leave Earth without the CTRL UL101.

[18] Sedlak P., Hirose Y., Enoki M. (2013). Acoustic emission localization in thin multi-layer plates using first-arrival determination. Mech. Syst. Signal Process..

[19] Kitajima A., Naohara N., Aihara A. (1984). Acoustic Leak Detection in Piping Systems.

[20] Holland S.D., Roberts R., Chimenti D.E., Song J.H. (2006). An ultrasonic array sensor for spacecraft leak direction finding. Ultransonics.

[21] Holland S.D., Roberts R., Chimenti D.E., Strei M. (2005). Leak detection in spacecraft using structure-borne noise with distributed sensors. Appl. Phys. Lett..

[22] Reusser R.S., Holland S.D., Roberts R.A., Chimenti D.E. (2007). Array-based acoustic leak location in spacecraft structures. AIP Conf. Proc..

[23] Mallet L., Lee B.C., Staszewski W.J., Scarpa F. (2004). Structural health monitoring using scanning laser vibrometry: II. Lamb waves for damage detection. Smart Mater. Struct..

[24] Daneshmand S., Sokhandan N., Zaeri-Amirani M., Lachapelle G. (2014). Precise Calibration of a GNSS Antenna Array for Adaptive Beamforming Applications. Sensors.

[25] Van Trees H.L., Harry L. (2002). Optimum Array Processing: Part IV of Detection, Estimation, and Modulation Theory.

[26] Hua Y., Sarkar T.K., Weiner D.D. (1991). An L-shaped array for estimating 2-D directions of wave arrival. IEEE Trans. Antennas Propag..

[27] Zhang X., Li J., Xu L. (2011). Novel two-dimensional DOA estimation with L-shaped array. EURASIP J. Adv. Signal Process..

[28] Schmidt R.O. (1986). Multiple emitter location and signal parameters estimation. IEEE Trans. Antennas Propag..

[29] Rose J.L. (2004). Ultrasonic Waves in Solid Media.

[30] Roberts R.A. (2010). Plate wave transmission/re-flection at geometric obstructions: Model study. AIP Conf. Proc..

[31] Holland S.D., Roberts R., Chimenti D.E., Strei M. (2005). Two-sensor ultrasonic spacecraft leak detection using structure-borne noise. Acoust. Res. Lett. Online.

